# Chemical Bonding and Dynamic Structural Fluxionality of a Boron-Based Na_5_B_7_ Sandwich Cluster

**DOI:** 10.3390/molecules28073276

**Published:** 2023-04-06

**Authors:** Peng-Fei Han, Ying-Jin Wang, Lin-Yan Feng, Shu-Juan Gao, Qiang Sun, Hua-Jin Zhai

**Affiliations:** 1 Nanocluster Laboratory, Institute of Molecular Science, Shanxi University, Taiyuan 030006, China; 2 Department of Chemistry, Xinzhou Teachers University, Xinzhou 034000, China; 3 Center for Applied Physics and Technology, School of Materials Science and Engineering, Peking University, Beijing 100871, China

**Keywords:** boron-based alloy clusters, three-layered sandwich cluster, multifold π/σ aromaticity, dynamic fluxionality, chemical bonding

## Abstract

Doping alkali metals into boron clusters can effectively compensate for the intrinsic electron deficiency of boron and lead to interesting boron-based binary clusters, owing to the small electronegativity of the former elements. We report on the computational design of a three-layered sandwich cluster, Na_5_B_7_, on the basis of global-minimum (GM) searches and electronic structure calculations. It is shown that the Na_5_B_7_ cluster can be described as a charge-transfer complex: [Na_4_]^2+^[B_7_]^3−^[Na]^+^. In this sandwich cluster, the [B_7_]^3−^ core assumes a molecular wheel in shape and features in-plane hexagonal coordination. The magic 6π/6σ double aromaticity underlies the stability of the [B_7_]^3−^ molecular wheel, following the (4*n* + 2) Hückel rule. The tetrahedral Na_4_ ligand in the sandwich has a [Na_4_]^2+^ charge-state, which is the simplest example of three-dimensional aromaticity, spherical aromaticity, or superatom. Its 2σ electron counting renders σ aromaticity for the ligand. Overall, the sandwich cluster has three-fold 6π/6σ/2σ aromaticity. Molecular dynamics simulation shows that the sandwich cluster is dynamically fluxional even at room temperature, with a negligible energy barrier for intramolecular twisting between the B_7_ wheel and the Na_4_ ligand. The Na_5_B_7_ cluster offers a new example for dynamic structural fluxionality in molecular systems.

## 1. Introduction

Due to the electron deficiency of boron with three valence electrons (2s^2^2p^1^), boron and its relevant compounds and clusters have rich and unique structures, as well as unconventional chemical bonding [1,2,3,4,5,6,7,8]. Recent experimental and theoretical studies have explored the structures and bonding of a wide range of boron-based clusters. It is now known that elemental boron clusters assume two-dimensional (2D) planar or quasi-planar geometries with up to 40 atoms for anions, extended 2D sheet structures (borophenes), and three-dimensional (3D) structures such as borospherenes [8,9,10,11,12,13]. Chemical bonding in these clusters is governed by π/σ aromaticity, antiaromaticity, and conflicting aromaticity, in which electron delocalization is essential in order to compensate for the intrinsic electron deficiency of boron.

Boron-based cluster nanomachines represent a new research direction in physical chemistry, in which small clusters demonstrate dynamic structural fluxionality [14,15,16,17,18,19,20,21,22,23,24,25,26,27,28,29,30,31]. Such unique dynamic behaviors are also dictated by boron’s electron deficiency. Researchers continuously designed and reported a series of pure boron clusters with dynamic fluxionality, such as B_19_^−^, B_13_^+^, B_18_^2−^, B_20_^−^, B_11_^−/0^, and B_15_^+^ [14,15,16,17,18,19,20,21,22]. In subsequent studies, researchers discovered that doping or mixing low electronegativity metals to form boron-based clusters is an effective way toward the rational design of metal-doped boron-based nanosystems with dynamic fluxionality [23,24,25,26,27,28,29,30,31]. For example, a binary Mg_2_B_8_ cluster was computationally designed as a “nanocompass”, in which an Mg_2_ needle was shown to rotate freely on the B_8_ baseplate [23]. By doping alkali metals to boron clusters, a series of boron-based sandwich clusters (B_7_Li_4_^+^, Na_6_B_7_^−^, and Na_8_B_7_^+^) were lately studied theoretically, which also show dynamic fluxionality [29,30]. In 2017, Zhai and coworkers discovered two virtually isoenergetic triple-layered and helix-type structures for a Be_6_B_11_^−^ cluster [31]. The former sandwich structure demonstrated dual dynamic rotation/twisting modes of structural fluxionality, akin to an earth–moon system at the nanoscale.

Alkali metal elements are clearly an ideal choice for doping due to their small electronegativities, which would allow precise tuning of the number of valence electrons in a boron-based alloy cluster, one at a time. The purpose of this work is to explore how the structure of a binary Na-B cluster depends on its ratio of Na versus B components, what underlies the stability of such a cluster structure, and whether new examples can be offered for dynamic structural fluxionality. To this end, we have reached a binary Na_5_B_7_ cluster. Computational global-minimum (GM) structure searches reveal a sandwich-type geometry for this cluster, which features a B_7_ molecular wheel sandwiched in between a Na_4_ tetrahedron and an isolated Na atom. Chemical bonding analysis suggests double 6π/6σ aromaticity for the B_7_ molecular wheel, as well as 2σ aromaticity for the tetrahedral Na_4_ ligand. In other words, the sandwich Na_5_B_7_ cluster has collectively three-fold 6π/6σ/2σ aromaticity. The sandwich shape allows the precise number of electrons to be transferred from the Na_4_ and Na ligands to the B_7_ core, so that the whole sandwich system can be stabilized via electrostatics. The cluster is faithfully formulated as [Na_4_]^2+^[B_7_]^3−^[Na]^+^; that is, a charge-transfer complex. The unique sandwich shape of the cluster facilitates dynamic structural fluxionality, as confirmed in molecular dynamics simulations. This work also highlights the robustness of [B_7_]^3−^ as a potential inorganic ligand.

## 2. Results

### 2.1. Global-Minimum and Transition-State Structures

**Figure 1:**
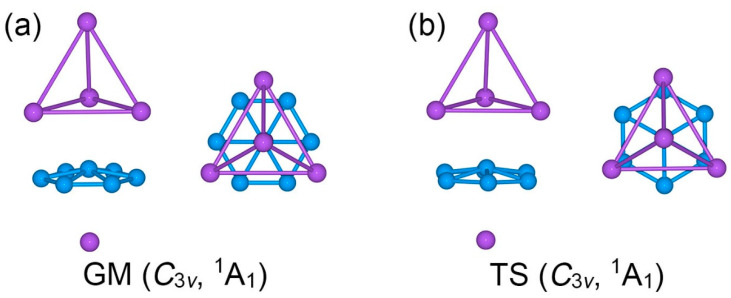
Optimized geometries of (**a**) *C*_3v_ (^1^A_1_) global minimum (GM) and (**b**) *C*_3v_ (^1^A_1_) transition state (TS) of Na_5_B_7_ cluster at the PBE0/6-311 + G* level. Both top- and side-views are presented.

The optimized low-lying structures of Na_5_B_7_ cluster at the PBE0/6-311 + G* level are shown in the Supplementary Materials (Figure S1). The GM Na_5_B_7_ cluster is identified herein using the global searches and electronic structure calculations at three levels of theory. The Cartesian coordinates for GM *C*_3v_ (^1^A_1_) Na_5_B_7_ cluster at the PBE0/6-311 + G* level are presented in Table S1. The PBE0/6-311 + G* method has been widely used for boron-based clusters [8,31,32]. The comparative B3LYP/6-311 + G* data for top candidate structures serve to check for the consistency of different functionals in terms of geometries and energetics. The single-point CCSD(T) calculations on the basis of optimized PBE0/6-311 + G* geometries allow benchmarking of the energetics. All three levels of theory confirm *C*_3v_ (^1^A_1_) structure (Figure 1a) as the GM of Na_5_B_7_ cluster.

Among the low-lying isomers is a local-minimum (LM) *C*_2v_ (^1^A_1_) structure (Figure S1), which is about 0.2 eV above the GM cluster. The remaining isomers are substantially higher in energy. Considering that adequate intramolecular charge transfers can take place from Na to B, the triplet-state structures are relatively unimportant for the present cluster system with an even number of 26 valence electrons. Indeed, our calculations show that the corresponding triplet-state structures for the top 12 singlet-state isomers in Figure S1 are 0.82–2.20 eV higher than the GM cluster at the PBE0/6-311 + G* level. Specifically, as illustrated in Figure S2, the lowest-lying triplet-state geometry of Na_5_B_7_ cluster, *C*_1_ (^3^A), lies 0.82 eV higher above the GM structure. Other triplet-state geometries are even higher in energy, by up to 2.20 eV (relative to the singlet GM cluster), for the 12th isomeric triplet structure. Thus, the *C*_3v_ (^1^A_1_) cluster (Figure 1a) is reasonably well defined on its potential energy surface as the real GM structure. Alternative structures, either singlets or triplets, are not energetically competitive, except for an LM *C*_2v_ (^1^A_1_) structure as mentioned above.

The top- and side-views of GM *C*_3v_ (^1^A_1_) Na_5_B_7_ cluster are presented in Figure 1a. Basically, it is a three-layered sandwich cluster. The boron component forms a quasi-planar B_7_ molecular wheel as the core layer of the sandwich. It features six-fold in-plane coordination for the central B site. On the other hand, the Na_5_ component is divided into a Na_4_ tetrahedron and an isolated Na atom. The two Na-based layers serve as ligands for the B_7_ molecular wheel. Specifically, three Na atoms in the Na_4_ tetrahedron are each situated on a B–B edge. The GM sandwich cluster may be referred to as the *staggered* conformation.

**Figure 2:**
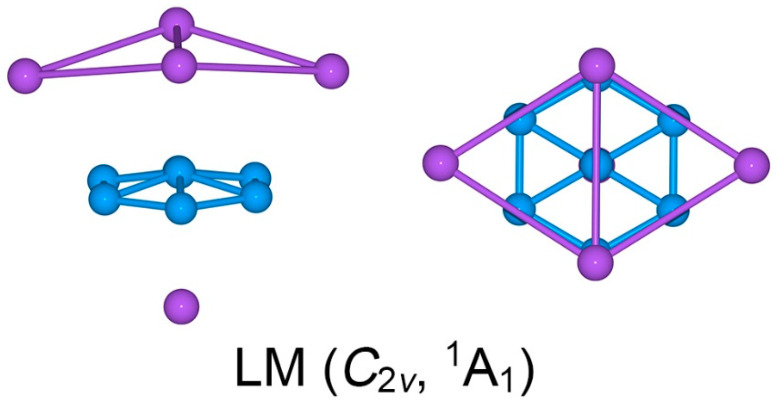
Optimized geometry of *C*_2v_ (^1^A_1_) local minimum (LM) of the Na_5_B_7_ cluster at the PBE0/6-311 + G* level. Both top- and side-views are presented.

Twisting the Na_4_ tetrahedron against the B_7_ molecular wheel by 30°, one readily reaches another *C*_3v_ (^1^A_1_) structure, which turns out to be a transition state (TS), as depicted in Figure 1b. The TS structure is referred to herein as the *eclipsed* conformation. The closest low-lying structure, LM *C*_2v_ (^1^A_1_), is also a sandwich (Figure 2). It differs from the GM structure in the top Na_4_ ligand. The Na_4_ ligand in the LM cluster is distorted into a roof-like (or rhombic) structure, so that only two Na atoms are coordinated to the B–B edges. The distortion alters the nature of bonding in the Na_4_ unit (*vide infra*).

### 2.2. Bond Distances, Wiberg Bond Indices, and Natural Atomic Charges

**Figure 3:**
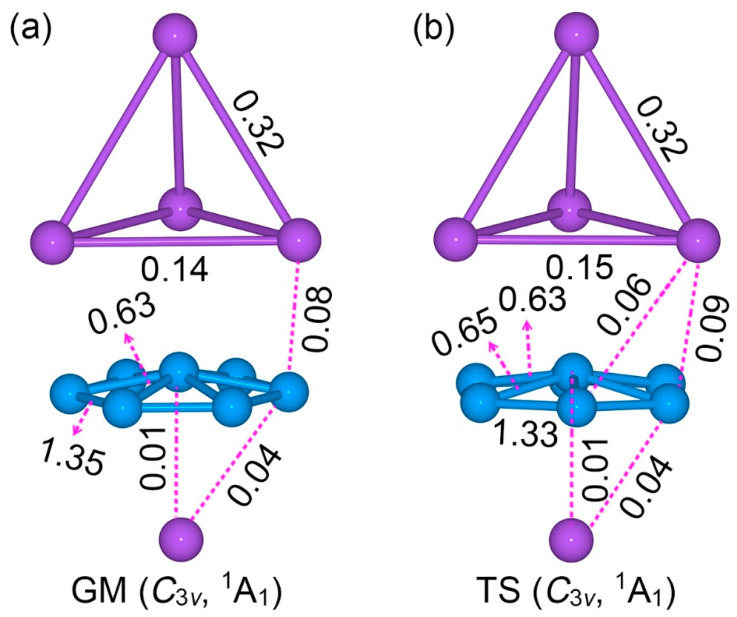
Wiberg bond indices (WBIs) for (**a**) GM and (**b**) TS structures of Na_5_B_7_ cluster at the PBE0/6-311 + G* level, as obtained from the natural bond orbital (NBO) analyses.

The calculated bond distances, Wiberg bond indices (WBIs), and natural atomic charges for GM and TS structures of the Na_5_B_7_ cluster are shown in Figure S3, Figure 3, and Figure S4, respectively. The three sets of data are generally coherent with each other. In particular, the GM and TS structures are quite similar, either qualitatively or quantitatively. Therefore, we shall primarily describe the GM cluster only.

In the GM cluster, the B_7_ wheel has uniform B–B distances for the peripheral links (1.61 Å) and radial ones (1.64 Å). These distances are shorter than the single bond (upper limit: 1.70 Å) [33]. It is invaluable to compare these with bare B_7_ cluster, whose peripheral and radial distances are 1.56–1.62 and 1.69–1.76 Å, respectively [34]. Thus, the peripheral B_6_ ring in GM Na_5_B_7_ cluster seems to be expanded, despite the fact that the radial B–B distances are clearly shortened. The above structural data are in line with a negatively charged B_7_ wheel in GM Na_5_B_7_ cluster.

The Na–Na distances for Na_4_ tetrahedron in the GM structure are 3.60/3.64 Å, whose shape also differs distinctly from that of a bare Na_4_ cluster. The latter assumes a rhombic *D*_2h_ structure [35]. The above Na–Na distances are far longer than a Na–Na single bond (3.10 Å) [33]. The tetrahedral Na_4_ ligand is not in a neutral state (*vide infra*). The shortest B–Na distance is 2.51 Å, which is longer than the recommended value for a B–Na σ single bond. The calculated WBIs fully support the above assignments (Figure 3a). The peripheral and radial B–B links have WBI values of 1.35 and 0.63, respectively. The Na–Na edges in the Na_4_ ligand have WBIs of 0.14–0.32, which are collectively consistent with a delocalized four-center two-electron (4c-2e) σ bond.

According to the above analysis, sandwich Na_5_B_7_ cluster has quite substantial intramolecular charge-transfers in between the B_7_ core and the Na_4_/Na ligands. The calculated natural atomic charges offer a quantitative picture (Figure S4a). Basically, each B atom in the B_7_ wheel has a negative charge from −0.27 to −0.35 |e|. Overall, the B_7_ wheel carries a total charge of −2.37 |e|. The tetrahedral Na_4_ ligand has a collective charge of +1.49 |e|, whereas the Na ligand carries a charge of +0.87 |e|. Clearly, the sandwich cluster can be formulated as a charge-transfer [Na_4_]^2+^[B_7_]^3−^[Na]^+^ complex. A similar analysis of the structure, WBIs, and natural atomic charges of the LM structure of Na_5_B_7_ cluster may be performed (see Figure S5).

We have also run structural optimization for a bare B_7_^3−^ trianion cluster at the PBE0/6-311 + G* level. The trianionic nature observed suggests that this should at most be considered as a model cluster. The interatomic distances may tend to expand to some extent, due to intramolecular Coulomb repulsion associated with three extra charges. The calculated bond distances and WBIs of the model B_7_^3−^ disk cluster are shown in Figure S6. The bond distances of the peripheral B–B links and radial ones are only 0.02 and 0.04 Å longer than those in the GM Na_5_B_7_ cluster, respectively. The WBIs of the peripheral B–B links and the radial ones show very minor variations relative to those in GM Na_5_B_7_ cluster, by 0.03 and 0.02, respectively. The central and peripheral B atoms in the model B_7_^3−^ cluster have negative charges of −0.30 and −0.45 |e|, respectively, which are close to those in the GM Na_5_B_7_ cluster. Overall, the central B atom in model B_7_^3−^ cluster is slightly above the B_6_ ring plane by 0.40 Å, as compared to 0.26 Å in GM Na_5_B_7_ cluster. The sandwich GM Na_5_B_7_ cluster moderately flattens the B_7_ wheel, which is intuitively expected and not surprising. The above comparison between the bare B_7_^3−^ model cluster and the B wheel in Na_5_B_7_ cluster further confirms the [B_7_]^3−^ nature of the B wheel in GM Na_5_B_7_ cluster. It is stressed that the [B_7_]^3−^ nature in GM Na_5_B_7_ cluster represents a solid conclusion on the basis of the structural data, natural bond orbital (NBO) analysis, and in particular the canonical molecular orbital (CMO) analysis and adaptive natural density partitioning (AdNDP) results. This conclusion stands firmly even without any calculations on a bare B_7_^3−^ model cluster. The latter calculations are merely an extra computational effort, which is a secondary part in the present paper.

## 3. Methods

The GM structure and low-lying isomers of Na_5_B_7_ cluster were explored by computer global searches using the Coalescence Kick (CK) algorithm [36,37], which were also aided by manual structure constructions. About 3000 stationary points were probed on the potential energy surface. The Gaussian 09 program was used subsequently to reoptimize the structures at the PBE0/6-311 + G* level [38,39,40]. To verify the reliability in terms of energetics, the top candidate structures were further assessed at the B3LYP/6-311 + G* and single-point CCSD(T)/6-311 + G*//PBE0/6-311 + G* levels [41]. Vibrational frequencies were calculated at the same density-functional theory (DFT) levels, that is, PBE0 and B3LYP, to ensure that the reported low-lying structures are true minima on the potential energy surface of the system, unless specifically stated otherwise.

Chemical bonding was elucidated using the CMO analysis, as well as the AdNDP analysis [42]. The AdNDP results were visualized using the Molekel 5.4.0.8 program [43]. The WBIs and charge distribution were calculated by NBO analysis [44] at the PBE0/6-311 + G* level. Born–Oppenheimer molecular dynamics (BOMD) simulations [45] were performed at PBE0/6-31G to demonstrate the structural fluxionality of the system.

## 4. Discussion

### 4.1. Chemical Bonding

**Figure 4:**
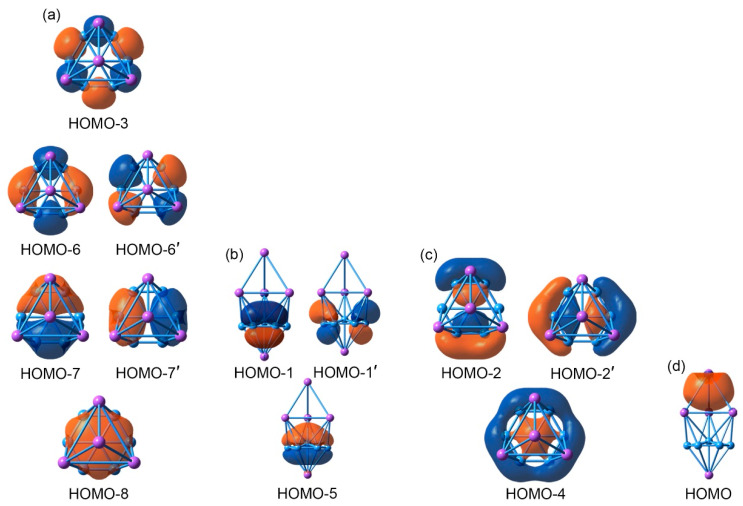
Pictures of the canonical molecular orbitals (CMOs) of GM *C*_3v_ (^1^A_1_) structure of the Na_5_B_7_ cluster. (**a**) Six σ CMOs for peripheral two-center two-electron (2c-2e) B–B Lewis σ bonds in the B_7_ wheel. (**b**) Three delocalized π CMOs. (**c**) Three delocalized σ CMOs. (**d**) One delocalized σ CMO over the Na_4_ tetrahedron. Subsets (**b**) through (**d**) collectively render three-fold 6π/6σ/2σ aromaticity for the cluster.

An in-depth chemical bonding analysis is essential toward understanding the stability, unique structure, and dynamic fluxionality of the title Na_5_B_7_ cluster. The CMO analysis is fundamental for this purpose. The Na_5_B_7_ cluster has 26 valence electrons. Their occupied CMOs are depicted in Figure 4. Of these 13 CMOs, six σ CMOs in subset (a) are primarily composed of B 2s atomic orbitals (AOs) of the peripheral B_6_ ring. These CMOs show from 0, 1, 2 to 3 nodal planes, including two degenerate pairs in the middle. Following the orbital construction principles, they can be recombined and localized as six Lewis-type two-center, two-electron (2c-2e) σ single bonds, one for each B–B edge.

Subset (b) in Figure 4 shows three π CMOs on the B_7_ wheel. Owing to the six-fold symmetry of the wheel, this π sextet cannot be localized as Lewis-type π bonds, akin to that in benzene, thus rendering π aromaticity for the sandwich cluster. The magic 6π electron counting conforms to the (4*n* + 2) Hückel rule. Likewise, the three σ CMOs in subset (c) have similar spatial distribution compared to those in the π sextet, except that the former CMOs are σ in nature. Again, the σ sextet is intrinsically delocalized and cannot be reduced to Lewis-type σ single bonds. It is, therefore, imperative to claim σ aromaticity for the sandwich as well, following the (4*n* + 2) Hückel rule.

Lastly, subset (d) shows only one σ CMO, which is clouded on the Na_4_ ligand. It is 4c-2e in nature and cannot be localized as one Lewis-type Na–Na σ single bond. The delocalized nature of these 2σ electrons renders three-dimensional σ aromaticity for the tetrahedral Na_4_ ligand and, hence, the sandwich cluster. Overall, the sandwich Na_5_B_7_ cluster collectively features three-fold π/σ aromaticity with the 6π/6σ/2σ electron counting.

**Figure 5:**
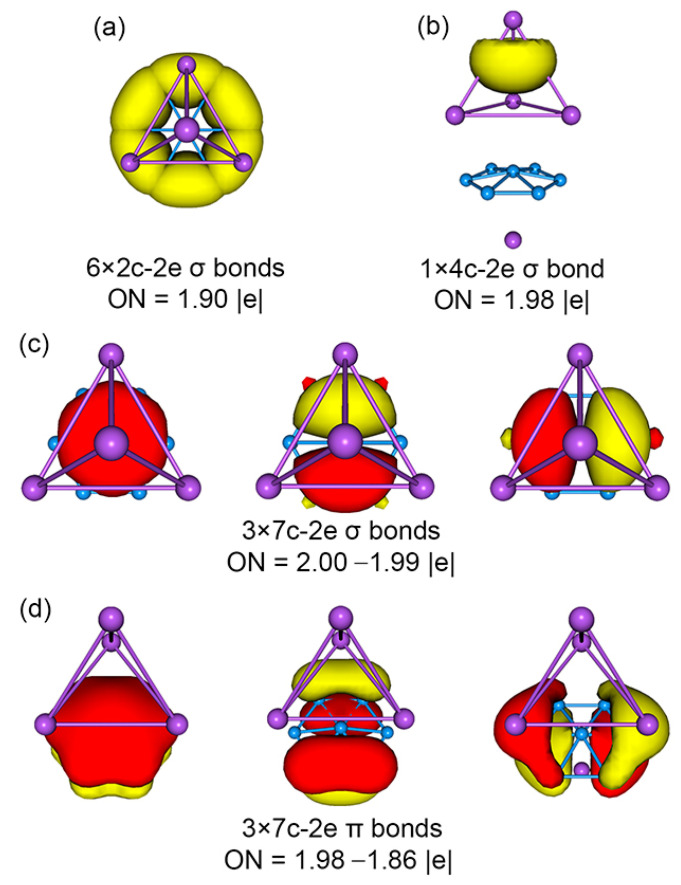
Chemical bonding scheme of *C*_3v_ (^1^A_1_) GM structure of the Na_5_B_7_ cluster on the basis of adaptive natural density partitioning (AdNDP) analysis. Occupation numbers (ONs) are shown.

The bonding picture is perfectly borne out from the AdNDP analysis. The AdNDP scheme of sandwich GM Na_5_B_7_ cluster is presented in Figure 5. The above analysis suggests that there is relatively minor covalent bonding between the three layers of the sandwich, and the cluster is indeed a charge-transfer complex. Similar CMO and AdNDP analyses can be conducted for the TS Na_5_B_7_ cluster, which are relatively straightforward and easy to understand. The relevant data are presented in Figures S7 and S8.

### 4.2. Dynamic Structural Fluxionality

The sandwich GM Na_5_B_7_ cluster, like numerous boron-based clusters reported in the recent literature [15,21,22,23,24,26,30,31,46,47], exhibits intriguing molecular dynamics properties. This is apparent on the basis of the close similarity between GM and TS structures in terms of the structures (Figure 3) and bonding (Figure 4 and Figure 5; Figures S7 and S8). In particular, the unique three-fold 6π/6σ/2σ aromaticity (see Section 4.1) underlies the dynamic fluxionality of GM Na_5_B_7_ cluster.

**Figure 6:**
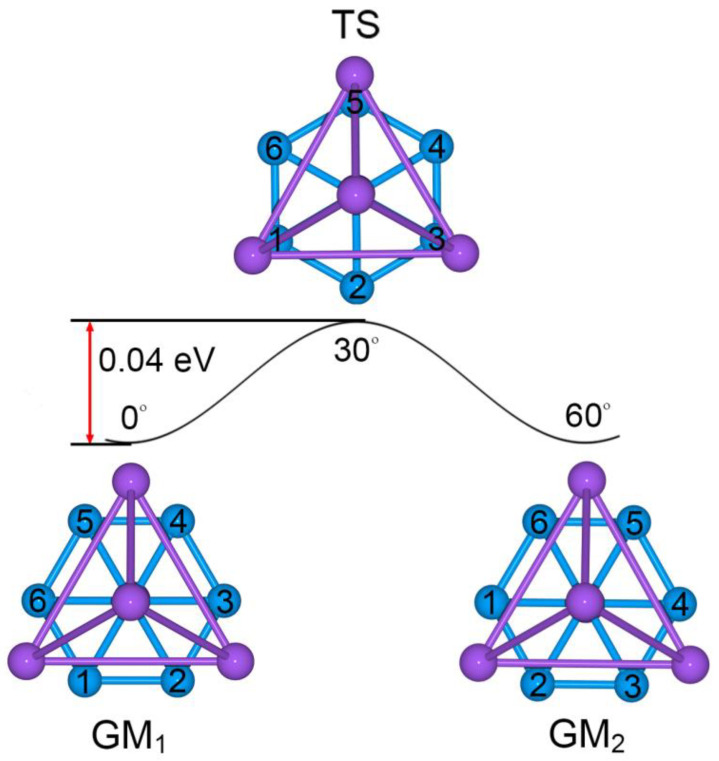
Structural evolution of Na_5_B_7_ cluster during the dynamic rotation. The B_7_ wheel is assumed to rotate clockwise with respect to the Na_4_ tetrahedron. The energy barrier is evaluated to be only 0.04 eV at the PBE0/6-311 + G* level.

**Figure 7:**
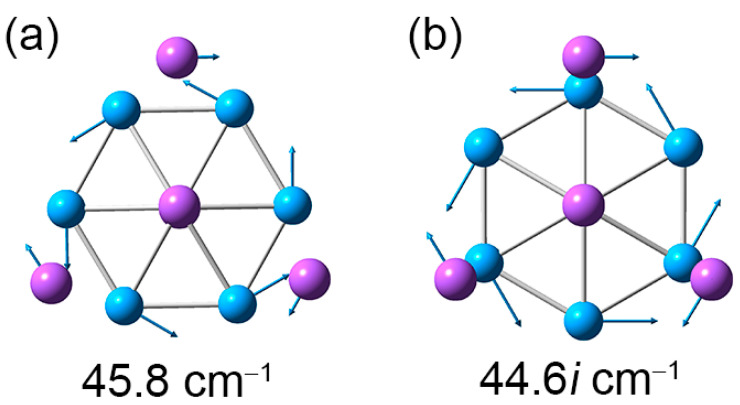
Displacement vectors for soft vibrational modes of Na_5_B_7_ cluster. (**a**) Soft mode for the GM structure. (**b**) Imaginary soft mode for the TS structure.

As shown in Figure 6, the GM and TS geometries of sandwich Na_5_B_7_ cluster are connected straightforwardly. Starting from GM (labeled as “GM_1_”) and twisting the B_7_ wheel clockwise relative to the Na_4_ tetrahedron by 30°, one readily reaches the TS structure. Further rotation in the same direction by 30° results in recovery of the GM structure (labeled as “GM_2_”). The energy barrier between the GM and TS structures is 0.04 eV at the PBE0/6-311 + G* level, which is relatively minor considering the anticipated strong electrostatic interaction between the B_7_ wheel and the Na_4_/Na ligands in the sandwich. The calculated soft vibrational modes of 45.8 and 44.6*i* cm^−1^, respectively, for the GM and TS structures are in line with the intramolecular rotation of the sandwich cluster (Figure 7).

To further validate the dynamic fluxionality of Na_5_B_7_ cluster, a BOMD simulation was performed at a selected temperature of 300 K. Specifically, the BOMD simulation was performed using the Hessian-based predictor-corrector method [48] at an initial setup temperature of 300 K, which was run for a time span of 50 ps (10,000 steps) at the PBE0/6-31G level. We took the GM geometry as the initial structure and simulated the dynamic evolution process. The vibrational sampling temperature was shown to be 298 K, which is close to the setting temperature. Before being recalculated analytically, the Bofill update method was used to update the Hessian evaluation for five steps. A trajectory step size of 1.0 amu^1/2^ bohr was used in the whole simulation process, and the total number of trajectories was 1. The maximum point for each trajectory was 200,000. Despite the variation in potential and kinetic energies during the BOMD process, the total energy remained constant, which was conserved to 10^−6^ hartree during the simulation process. A short movie extracted from the BOMD data is presented in the Supplementary Materials, which vividly demonstrates the intriguing structural dynamics of the cluster (see Video S1). In short, the title cluster is dynamically fluxional even at near room temperature. We comment here that the BOMD simulation represents a relatively minor part in this study. It is not intended to provide any information with accuracy. It offers molecular dynamics information of the system qualitatively, rather than quantitatively.

### 4.3. On the Low-Lying LM Structure: The Importance of Three-Fold π/σ Aromaticity for GM Na_5_B_7_ Cluster

Our exploration of the potential energy surface of Na_5_B_7_ cluster shows that only two structures, GM (*C*_3v_, ^1^A_1_) and LM (*C*_2v_, ^1^A_1_), are close in energy, within about 0.2 eV at all three levels of theory (Figure S1). Other isomeric structures are substantially higher in energy, by at least 0.9 eV. Therefore, it is invaluable to understand the LM cluster, which is depicted in Figure 2. Basically, the LM and GM structures differ in the upper Na_4_ ligand. While the Na_4_ ligand in GM cluster is a slightly distorted tetrahedron (3.60 versus 3.64 Å; Figure S3a), it becomes a roof-like, quasi-planar ligand (or a rhombic ligand) in the LM cluster (Figure S5a). In the LM cluster, the roof-like Na_4_ ligand appears to interact with the B_7_ wheel primarily via two Na atoms along the longer diagonal, which allows optimal intramolecular charge transfer (Figure S5c). As a consequence, chemical bonding in the roof-like Na_4_ ligand is dominated by σ bonding along the shorter diagonal. The shorter Na_2_ diagonal in the highest occupied molecular orbital (HOMO) contributes to 66% of the whole Na_4_ ligand (Figures S9d and S10b). In other words, chemical bonding in the roof-like Na_4_ ligand is largely a Lewis-type 2c-2e σ bond in nature, at least in the zeroth-order picture.

In contrast, the 2σ framework in GM cluster is truly delocalized in a three-dimensional manner, which offers extra stabilization to the sandwich GM cluster. As a model system, one can evaluate a free-standing Na_4_^2+^ dication cluster in its tetrahedral *T*_d_ and rhombic *D*_2h_ geometries (Figure S11). At the PBE0 level, the *T*_d_ cluster is 0.21 eV more stable than its rhombic *D*_2h_ isomer. This energetic difference is comparable to those between the GM and LM structures of Na_5_B_7_ cluster (Figure S1). Thus, 2σ aromaticity is clearly a decisive factor that helps distinguish between the GM Na_5_B_7_ cluster from its LM isomer.

The tetrahedral Na_4_ ligand, in its specific [Na_4_]^2+^ charge state, is the simplest polyhedral structure. Hence it can be considered the simplest example of “spherical” aromaticity. The 2σ electron counting also conforms to the 2(*n* + 1)^2^ rule for spherical aromaticity. Alternatively, the tetrahedral Na_4_ ligand may be viewed as the simplest superatom [49,50,51].

**Figure 8:**
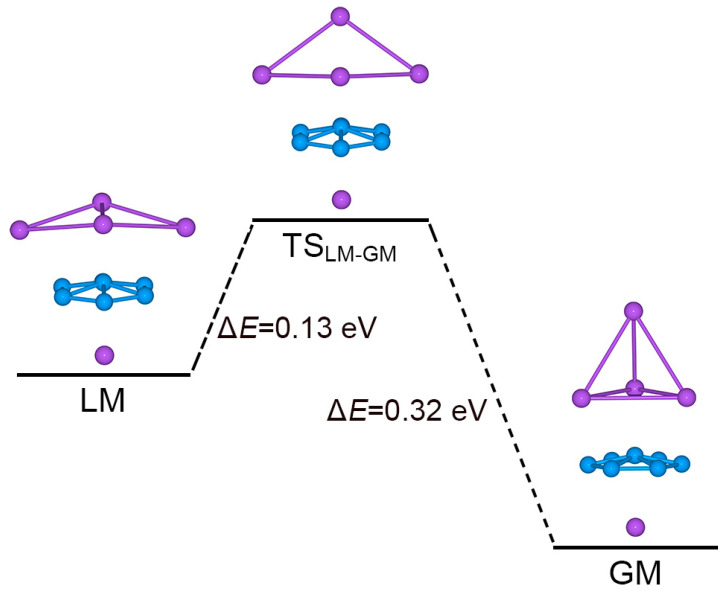
Evolution from the LM structure to the GM structure of Na_5_B_7_ cluster. The TS_LM-GM_ structure is a transition state that connects LM and GM. Relative energies are shown in eV at the PBE0/6-311 + G* level.

**Figure 9:**
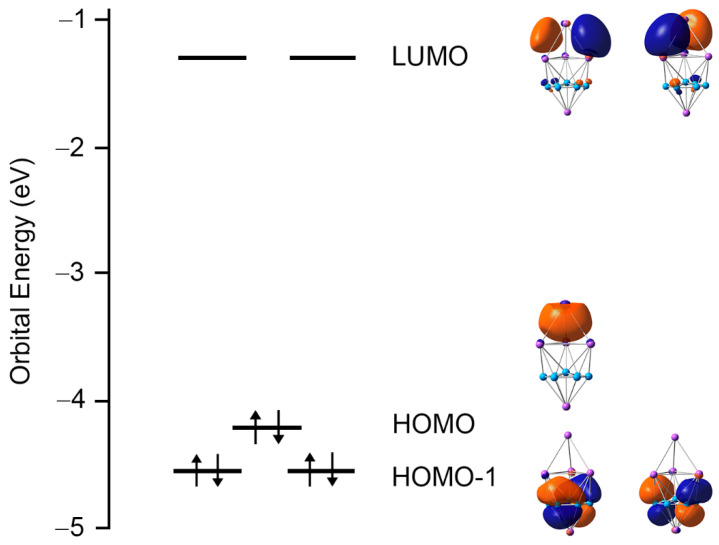
Orbital energy diagram of GM Na_5_B_7_ cluster at the PBE0/6-311 + G* level.

Interestingly, one can directly connect the GM and LM structures via a TS geometry, TS_LM–GM_, as shown in Figure 8. The TS_LM–GM_ is located at only 0.13 eV above LM at PBE0. The TS_LM–GM_ structure has a soft imaginary mode of 53.0*i* cm^−1^. The GM cluster lies 0.19 eV below LM, as well as 0.32 eV below TS_LM–GM_. According to this energy diagram, the GM cluster is relatively robust against isomerization. Our electronic structure calculations also indicate that the energy gap of GM Na_5_B_7_ cluster (2.89 eV; see Figure 9), between its HOMO and lowest unoccupied molecular orbital (LUMO), is significantly larger than that in its LM isomer (0.25 eV), thus demonstrating the electronic robustness of sandwich GM Na_5_B_7_ cluster. Indeed, the sizable energy gap in Figure 9 is a strong indication that a [B_7_]^3−^ motif in binary Na_5_B_7_ cluster is appropriate, which favors a closed-shell configuration in the triply charged anionic state. The same reason underlies the nature of a charge-transfer [Na_4_]^2+^[B_7_]^3−^[Na]^+^ complex for GM Na_5_B_7_ cluster, as well as its unique sandwich geometry.

## 5. Conclusions

We have computationally designed a boron-based alloy Na_5_B_7_ cluster that assumes unique three-layered sandwich structure. The cluster may be described as a charge-transfer [Na_4_]^2+^[B_7_]^3−^[Na]^+^ complex, which is composed of a quasi-planar B_7_ wheel as core, as well as a Na_4_ tetrahedron and an isolated Na atom as its two ligand layers at the top and at the bottom. Chemical bonding analysis indicates magic 6π/6σ double aromaticity for the [B_7_]^3−^ wheel and three-dimensional 2σ aromaticity for the tetrahedral [Na_4_]^2+^ ligand. The latter is also the simplest example of three-dimensional or spherical aromaticity, as well as the simplest superatom. Collectively, the Na_5_B_7_ cluster has three-fold 6π/6σ/2σ aromaticity, which underlies its thermodynamic stability. The same mechanism facilitates the intriguing dynamic structural fluxionality for the sandwich cluster, even at near room temperature. This work also highlights the structural and electronic robustness of the [B_7_]^3−^ molecular wheel as a potential inorganic ligand.

## Data Availability

Not applicable.

## Supplementary Materials

The following supporting information can be downloaded at: https://www.mdpi.com/article/10.3390/molecules28073276/s1. Table S1: Cartesian coordinates for global-minimum (GM) and transition-state (TS) structures of Na_5_B_7_ cluster at PBE0/6-311 + G*; Figure S1: Alternative optimized structures of Na_5_B_7_ cluster at PBE0; Figure S2: Selected triplet-state structures of Na_5_B_7_ cluster; Figures S3 and S4: Calculated bond distances and natural atomic charges for GM and TS structures of Na_5_B_7_ cluster; Figure S5: Bond distances, Wiberg bond indices (WBIs), and natural atomic charges for a local minimum (LM) structure; Figure S6: Calculated bond distances and WBIs of model B_7_^3−^ cluster at PBE0/6-311 + G*; Figures S7–S10: CMOs and AdNDP schemes of TS and LM structures of Na_5_B_7_ cluster; Figure S11: Relative energies for tetrahedral versus rhombic structures of a free-standing Na_4_^2+^ model cluster at PBE0; Video S1: A short movie extracted from the BOMD simulation for Na_5_B_7_ cluster at 300 K.
