# Supplementary material for: Chemical Bonding and Dynamic Structural Fluxionality of a Boron-Based Na5B7 Sandwich Cluster
Source: Molecules. 2023 Apr 6;28(7):3276. doi: 10.3390/molecules28073276 (PMC10096537; doi:10.3390/molecules28073276)

SUPPLEMENTARY MATERIALS

Chemical bonding and dynamic structural fluxionality of a boron-based Na_5B_7 sandwich cluster

Peng-Fei Han,[1] Ying-Jin Wang,[2] Lin-Yan Feng,[1,2] Shu-Juan Gao,[1] Qiang Sun,[3] and Hua-Jin Zhai[1,*]

[1]*Nanocluster Laboratory, Institute of Molecular Science, Shanxi University, Taiyuan 030006, China*

[2]*Department of Chemistry, Xinzhou Teachers University, Xinzhou 034000, China*

[3]*Center for Applied Physics and Technology, School of Materials Science and Engineering, Peking University, Beijing 100871, China*

*E-mail: hj.zhai@sxu.edu.cn

Table S1. Cartesian coordinates for the global-minimum (GM) and transition state (TS) structures, C_{3v} ($^1A_1$), of Na_5B_7 cluster at the PBE0/6-311+G* level.

Figure S1. Alternative optimized low-lying structures of Na_5B_7 cluster at the PBE0/6-311+G* level. Relative energies are shown without brackets at PBE0, including corrections for zero-point energies (ZPEs). Also shown are energies for top low-lying isomers at the single-point CCSD(T)/6-311+G* (in square brackets) and B3LYP/6-311+G* (in brackets) levels. All energies are in eV.

Figure S2. Geometries and energetics of the first 12 triplet-state structures of Na_5B_7 cluster at the PBE0/6-311+G* level. Relative energies are shown in eV with respect to the GM structure.

Figure S3. Calculated bond distances (in Å) for (a) GM C_{3v} ($^1A_1$) and (b) TS C_{3v} ($^1A_1$) structures of Na_5B_7 cluster at the PBE0/6-311+G* level.

Figure S4. Calculated natural atomic charges in |e| for (a) GM and (b) TS structures of Na_5B_7 cluster at the PBE0/6-311+G* level, as obtained from the natural bond orbital (NBO) analyses.

Figure S5. Calculated (a) bond distances (in Å), (b) Wiberg bond indices (WBIs), and (c) natural atomic charges (in |e|) for a local-minimum (LM) C_{2v} ($^1A_1$) structure of Na_5B_7 cluster.

Figure S6. Calculated bond distances (black color, in Å) and WBIs (in pink) of a bare C_{6v} B_7^{3-} model cluster at the PBE0/6-311+G* level.

Figure S7. Pictures of canonical molecular orbitals (CMOs) of the TS C_{3v} ($^1A_1$) structure of Na_5B_7 cluster. (a) Six σ CMOs for peripheral two-center two-electron (2c-2e) B−B σ single bonds in the B_7 wheel. (b) Three delocalized π CMOs. (c) Three delocalized σ CMOs. (d) One delocalized σ CMO for the top Na_4 tetrahedron. Subsystems (b) through (d) collectively render three-fold 6π/6σ/2σ aromaticity for TS C_{3v} ($^1A_1$) structure.

Figure S8. Chemical bonding scheme of the TS C_{3v} ($^1A_1$) structure for Na_5B_7 cluster on the basis of adaptive natural density partitioning (AdNDP) analysis. Occupation numbers (ONs) are indicated.

Figure S9. The CMO pictures of a LM C_{2v} ($^1A_1$) structure of Na_5B_7 cluster. (a) Six σ CMOs for peripheral 2c-2e B−B σ single bonds in the B_7 wheel. (b) Three delocalized π CMOs. (c) Three delocalized σ CMOs. (d) One σ CMO over the rhombic Na_4 unit.

Figure S10. Chemical bonding scheme of a LM C_{2v} ($^1A_1$) structure of Na_5B_7 cluster on the basis of AdNDP analysis. The ONs are indicated.

Figure S11. Relative energies (in eV) for tetrahedral versus rhombic structures of a free-standing Na_4^{2+} model cluster at the PBE0/6-311+G* level.

Table S1. Cartesian coordinates for the global-minimum (GM) and transition state (TS) structures, C_{3v} ($^1A_1$), of Na_5B_7 cluster at the PBE0/6-311+G* level.

GM, Na_5B_7 (C_{3v}, $^1A_1$)

B	-1.61375400	0.00051400	-1.29306500
B	-0.80643100	1.40566809	-1.29306500
B	0.80732200	1.39729500	-1.29306500
B	1.61375400	0.00051400	-1.29306500
B	0.80643100	-1.39780900	-1.29306500
B	-0.80732200	1.39729500	-1.29306500
B	0.00000000	0.00000000	-0.98016900
Na	0.00000000	2.10180900	1.13529800
Na	0.00000000	0.00000000	4.05349300
Na	-1.82022000	-1.05090400	1.13529800
Na	1.82022000	-1.05090400	1.13529800
Na	0.00000000	0.00000000	-3.48731400

TS, Na_5B_7 (C_{3v}, $^1A_1$)

B	-1.39887500	0.80764100	-1.27191500
B	-1.39553100	-0.80571000	-1.32879900
B	1.39887500	0.80764100	-1.27191500
B	1.39553100	-0.80571000	-1.32879900
B	0.00000000	-1.61528200	-1.27191500
B	0.00000000	1.61142000	-1.32879900
B	0.00000000	0.00000000	-0.99539300

Na	0.00000000	2.06979600	1.14197700
Na	0.00000000	0.00000000	4.07153500
Na	-1.79249600	-1.03489800	1.14197700
Na	1.79249600	-1.03489800	1.14197700
Na	0.00000000	0.00000000	-3.49858600

Figure S1. Alternative optimized low-lying structures of Na_5B_7 cluster at the PBE0/6-311+G* level. Relative energies are shown without brackets at PBE0, including corrections for zero-point energies (ZPEs). Also shown are energies for top low-lying isomers at the single-point CCSD(T)/6-311+G* (in square brackets) and B3LYP/6-311+G* (in brackets) levels. All energies are in eV.

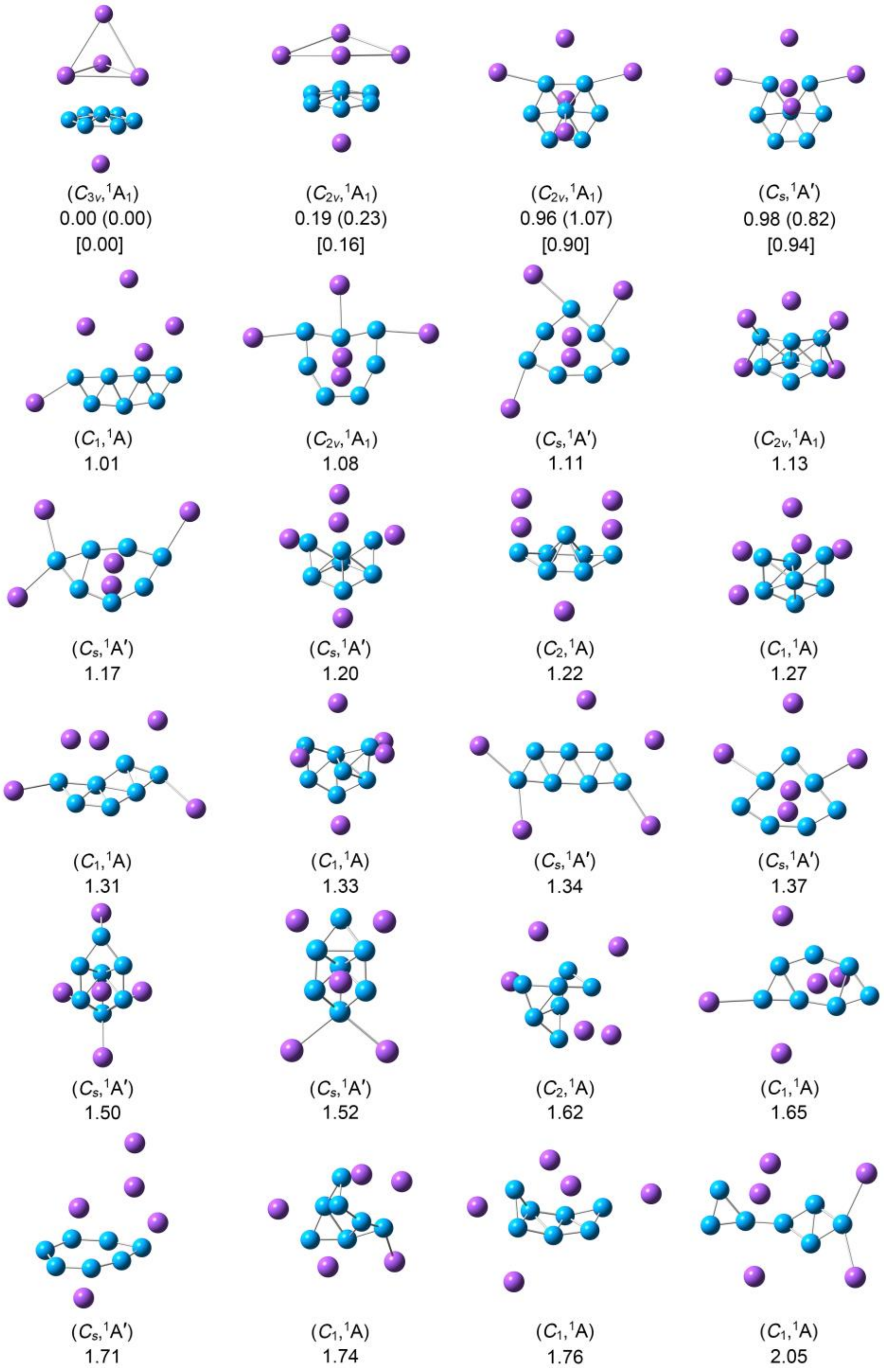

Figure S2. Geometries and energetics of the first 12 triplet-state structures of Na_5B_7 cluster at the PBE0/6-311+G* level. Relative energies are shown in eV with respect to the GM structure.

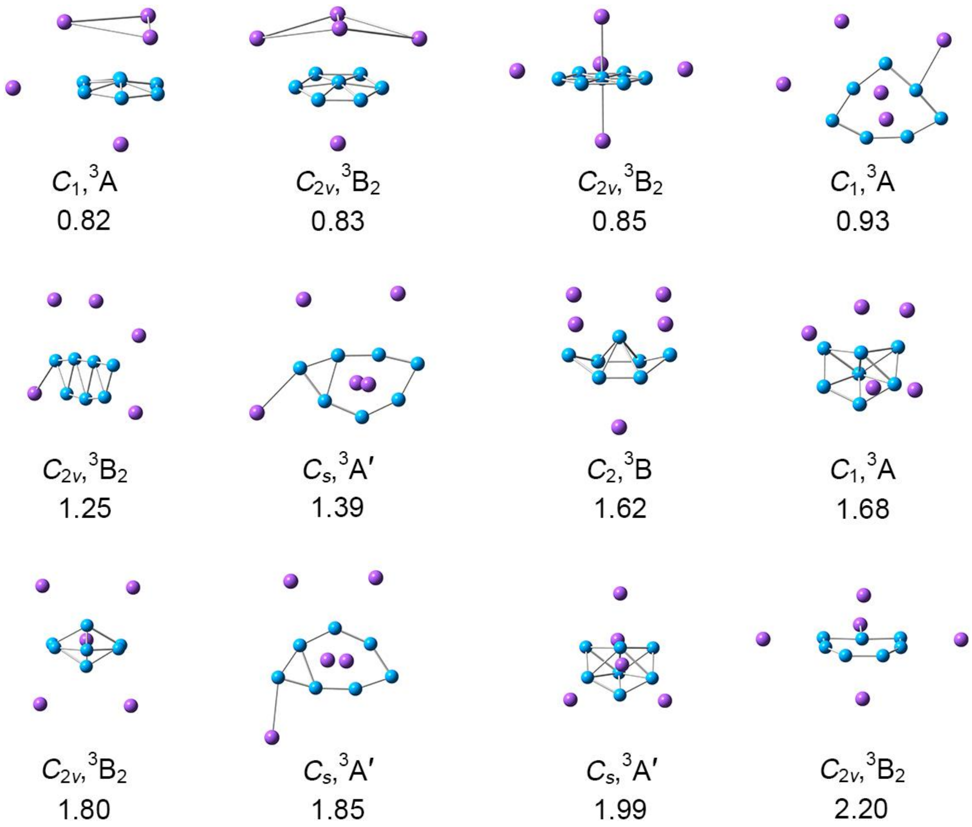

Figure S3. Calculated bond distances (in Å) for (a) GM C_{3v} ($^1A_1$) and (b) TS C_{3v} ($^1A_1$) structures of Na_5B_7 cluster at the PBE0/6-311+G* level.

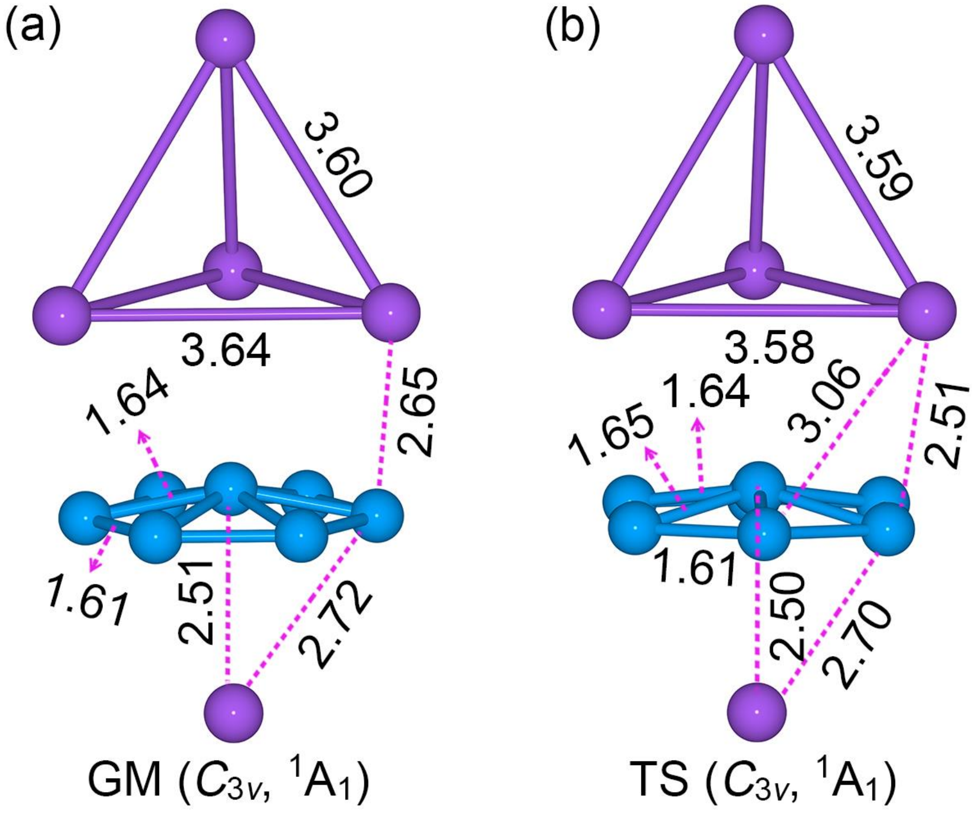

Figure S4. Calculated natural atomic charges in |e| for (a) GM and (b) TS structures of Na_5B_7 cluster at the PBE0/6-311+G* level, as obtained from the natural bond orbital (NBO) analyses.

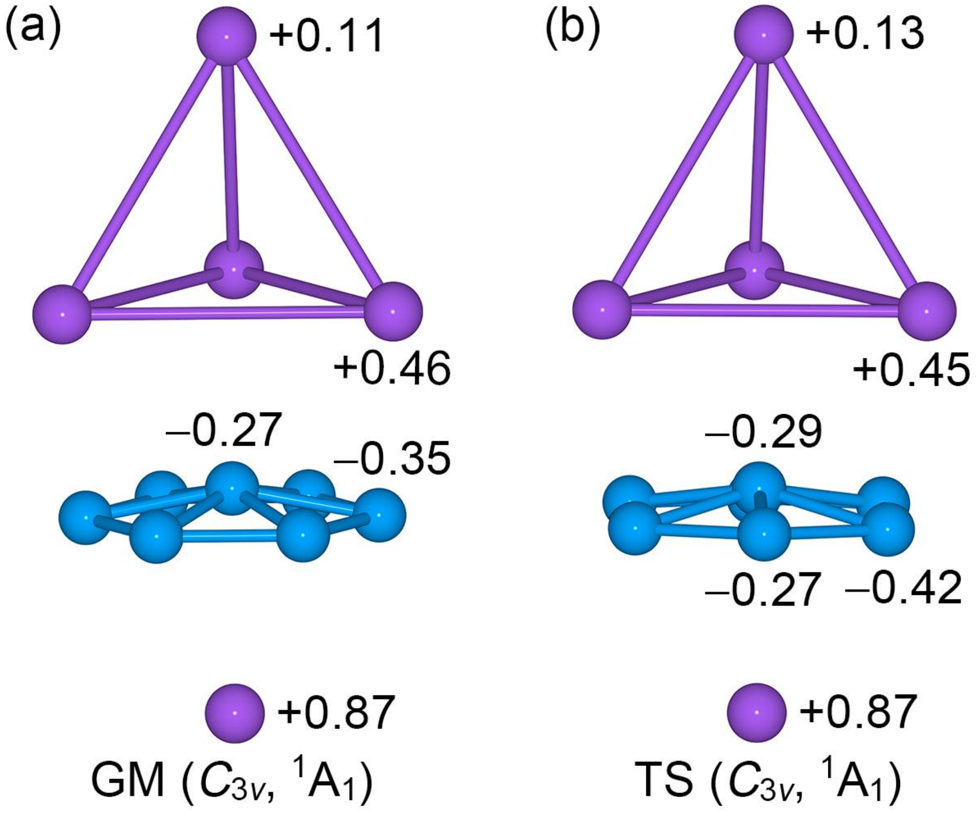

Figure S5. Calculated (a) bond distances (in Å), (b) Wiberg bond indices (WBIs), and (c) natural atomic charges (in |e|) for a local-minimum (LM) C_{2v} ($^1A_1$) structure of Na_5B_7 cluster.

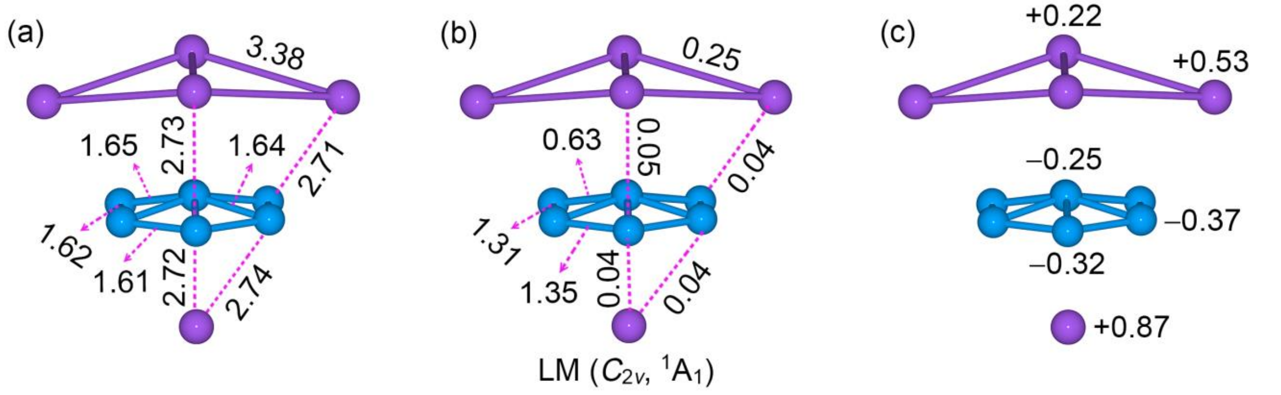

Figure S6. Calculated bond distances (black color, in Å) and WBIs (in pink) of a bare C_{6v} B_7^{3-} model cluster at the PBE0/6-311+G* level.

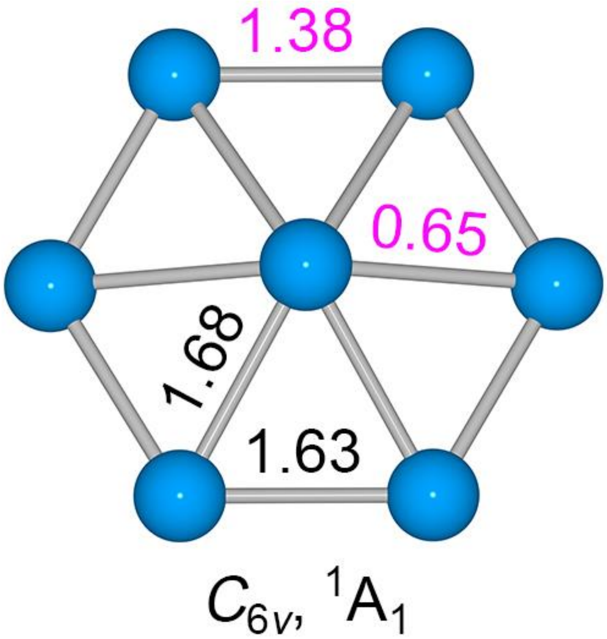

Figure S7. Pictures of canonical molecular orbitals (CMOs) of the TS C_{3v} ($^1A_1$) structure of Na_5B_7 cluster. (a) Six σ CMOs for peripheral two-center two-electron (2c-2e) B–B σ single bonds in the B_7 wheel. (b) Three delocalized π CMOs. (c) Three delocalized σ CMOs. (d) One delocalized σ CMO for the top Na_4 tetrahedron. Subsystems (b) through (d) collectively render three-fold 6π/6σ/2σ aromaticity for TS C_{3v} ($^1A_1$) structure.

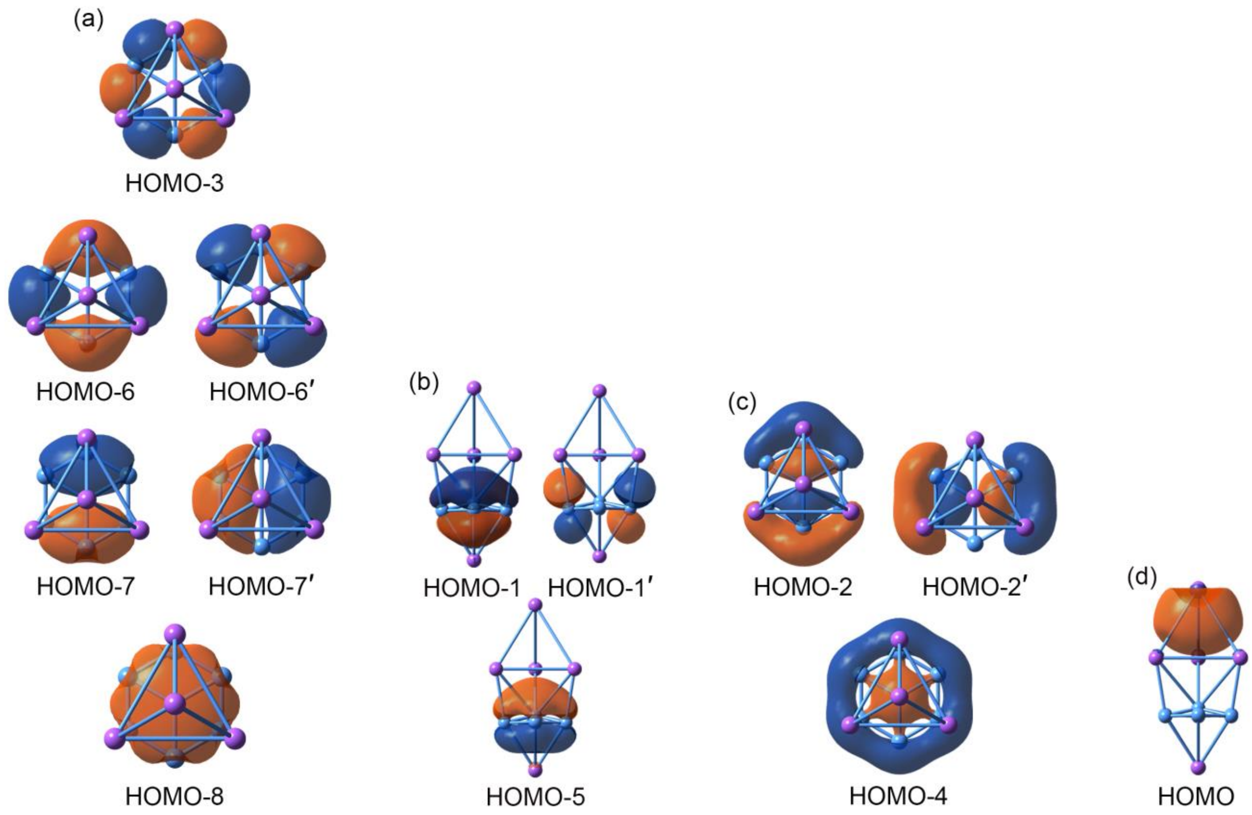

Figure S8. Chemical bonding scheme of the TS C_{3v} ($^1A_1$) structure for Na_5B_7 cluster on the basis of adaptive natural density partitioning (AdNDP) analysis. Occupation numbers (ONs) are indicated.

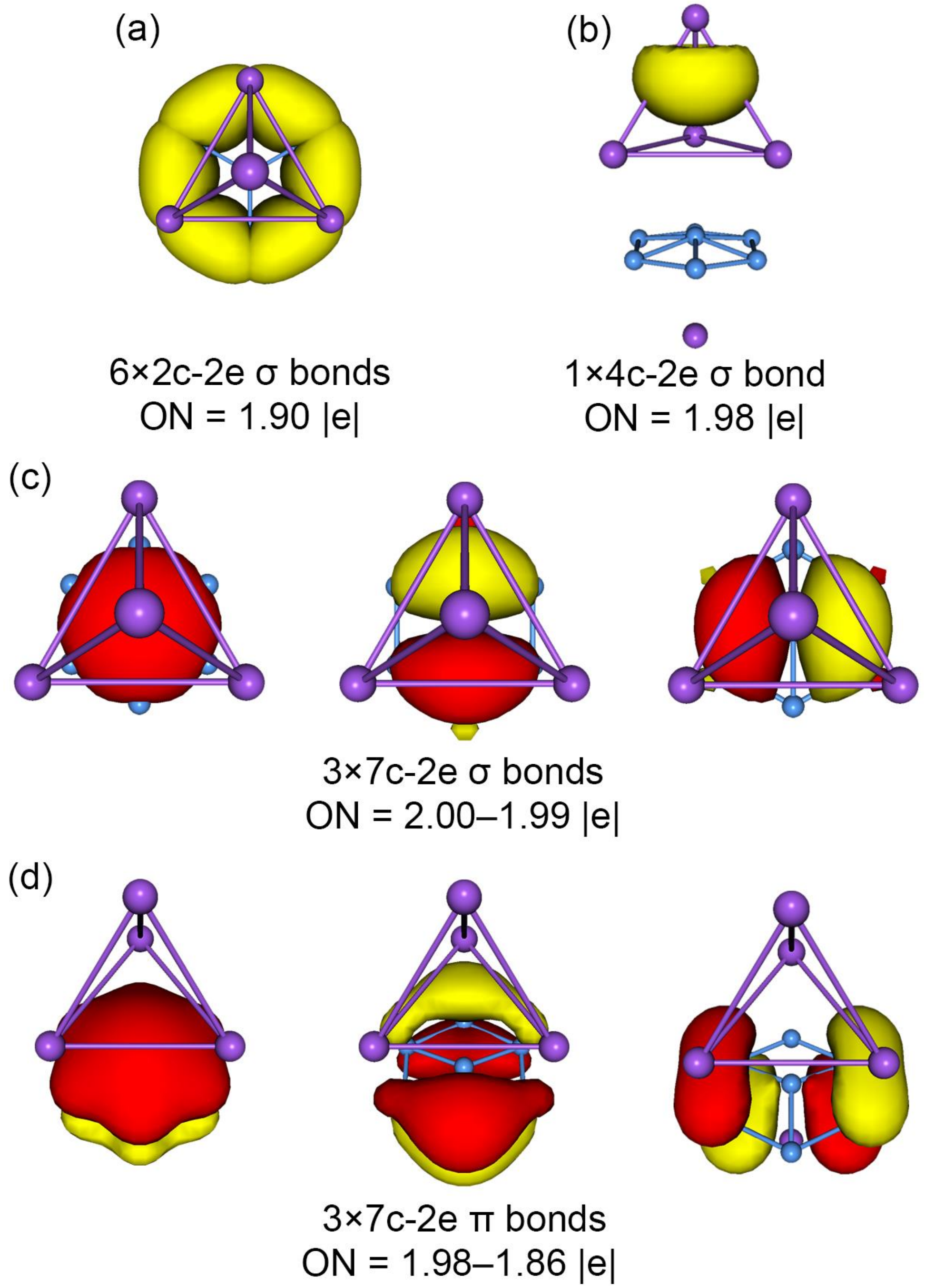

Figure S9. The CMO pictures of a LM C_{2v} ($^1A_1$) structure of Na_5B_7 cluster. (a) Six σ CMOs for peripheral 2c-2e B–B σ single bonds in the B_7 wheel. (b) Three delocalized π CMOs. (c) Three delocalized σ CMOs. (d) One σ CMO over the rhombic Na_4 unit.

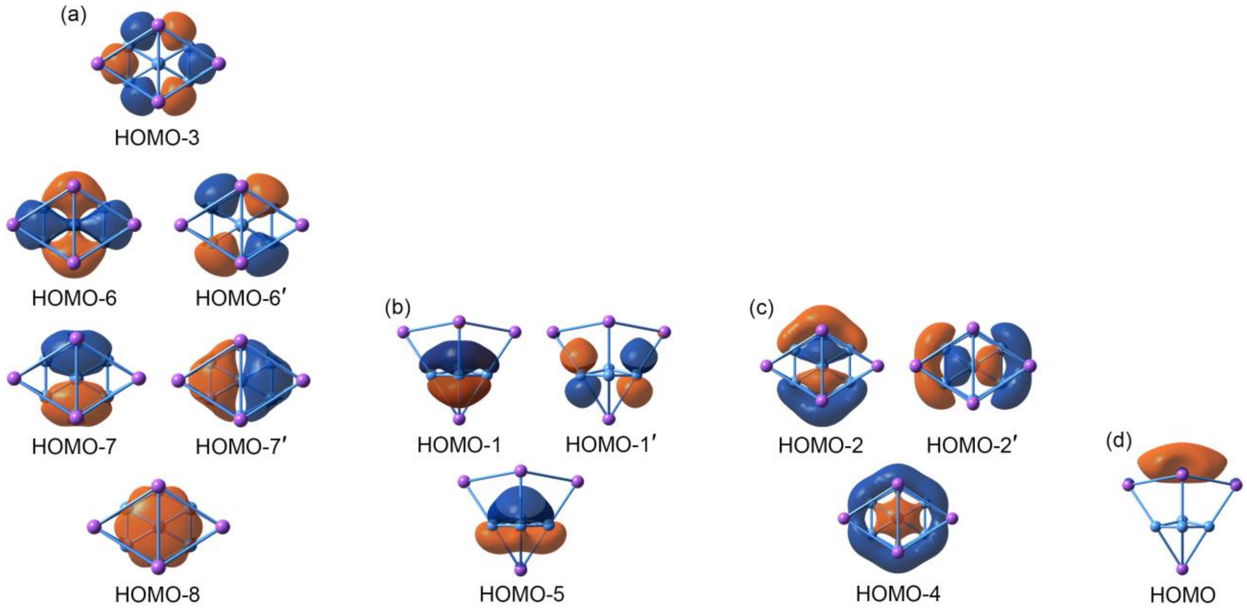

Figure S10. Chemical bonding scheme of a LM C_{2v} ($^1A_1$) structure of Na_5B_7 cluster on the basis of AdNDP analysis. The ONs are indicated.

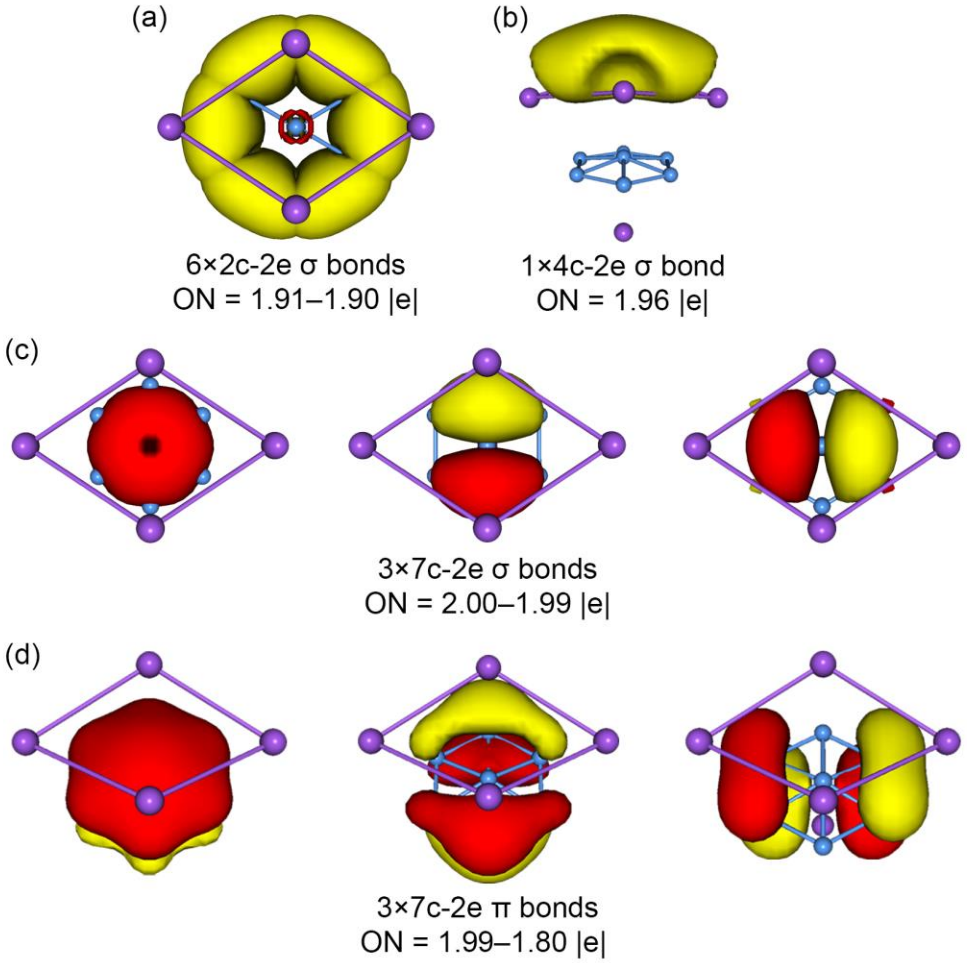

Figure S11. Relative energies (in eV) for tetrahedral versus rhombic structures of a free-standing Na_4^{2+} model cluster at the PBE0/6-311+G* level.

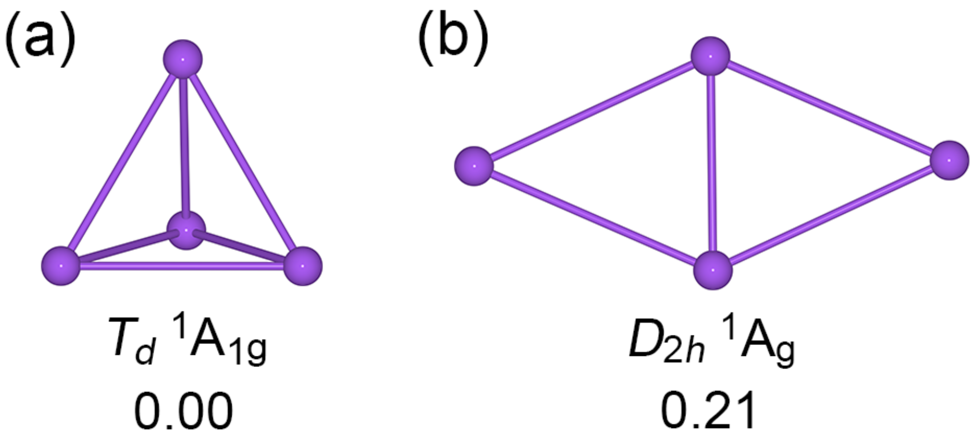

Supplement: Supplementary file 1 [file molecules-28-03276-s001.zip › R1_Molecules_dynamic_B7Na5_ESI.pdf]
