# Supplementary material for: Chemical Bonding and Dynamic Structural Fluxionality of a Boron-Based Na5B7 Sandwich Cluster
Source: Molecules. 2023 Apr 6;28(7):3276. doi: 10.3390/molecules28073276 (PMC10096537; doi:10.3390/molecules28073276)
Supplement: Supplementary file 1 [file molecules-28-03276-s001.zip › R1_Molecules_dynamic_B7Na5_TOC.pdf]

Table of Contents:

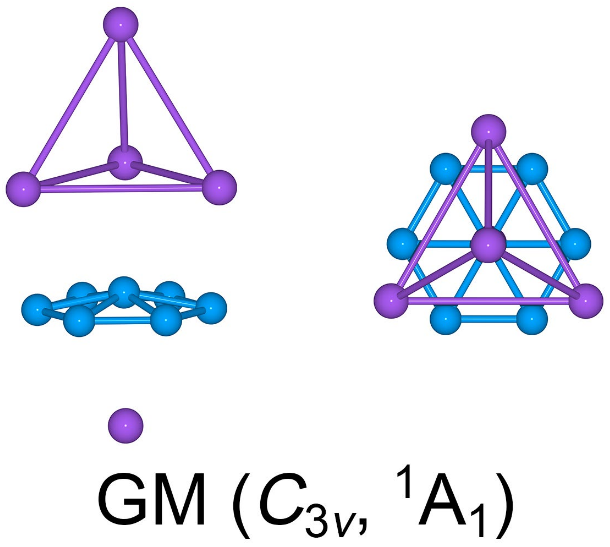

A three-layered charge-transfer $[Na_4]^{2+}[B_7]^{3-}[Na]^+$ cluster has three-fold $6\pi/6\sigma/2\sigma$ aromaticity. Its tetrahedral $[Na_4]^{2+}$ ligand is a simple example of three-dimensional aromaticity or superatom.
